# The palatability of sugar-sweetened beverage taxation: A content analysis of newspaper coverage of the UK sugar debate

**DOI:** 10.1371/journal.pone.0207576

**Published:** 2018-12-05

**Authors:** Christina H. Buckton, Chris Patterson, Lirije Hyseni, S. Vittal Katikireddi, Ffion Lloyd-Williams, Alex Elliott-Green, Simon Capewell, Shona Hilton

**Affiliations:** 1 MRC/CSO Social and Public Health Sciences Unit, Institute of Health and Wellbeing, University of Glasgow, Glasgow, United Kingdom; 2 Department of Public Health & Policy, University of Liverpool, Liverpool, United Kingdom; McMaster University, CANADA

## Abstract

**Background:**

Excess sugar consumption, including sugar-sweetened beverages (SSBs), contributes to a variety of negative health outcomes, particularly for young people. The mass media play a powerful role in influencing public and policy-makers’ perceptions of public health issues and their solutions. We analysed how sugar and SSB policy debates were presented in UK newspapers at a time of heightened awareness and following the announcement of the UK Government’s soft drinks industry levy (SDIL), to inform future public health advocacy.

**Methods & findings:**

We carried out quantitative content analysis of articles discussing the issues of sugar and SSB consumption published in 11 national newspapers from April 2015 to November 2016. 684 newspaper articles were analysed using a structured coding frame. Coverage peaked in line with evidence publication, campaigner activities and policy events. Articles predominantly supportive of SSB taxation (23.5%) outnumbered those that were predominantly oppositional (14.2%). However, oppositional articles outnumbered supportive ones in the month of the announcement of the SDIL. Sugar and SSB consumption were presented as health risks, particularly affecting young people, with the actions of industry often identified as the cause of the public health problem. Responsibility for addressing sugar overconsumption was primarily assigned to government intervention.

**Conclusion:**

Our results suggest that the policy landscape favouring fiscal solutions to curb sugar and SSB consumption has benefited from media coverage characterising the issue as an industry-driven problem. Media coverage may drive greater public acceptance of the SDIL and any future taxation of products containing sugar. However, future advocacy efforts should note the surge in opposition coinciding with the announcement of the SDIL, which echoes similar patterns of opposition observed in tobacco control debates.

## Introduction

Excess consumption of sugary foods and sugar-sweetened beverages (SSBs), particularly among children and adolescents, is a major public health concern. Sugar contributes to obesity, which in turn increases the risk factors for type-2 diabetes and other non-communicable diseases such as cardiovascular disease and common cancers [1–4]. Excess sugar consumption is also associated with poor dental health [5, 6]. In 2015, approximately 27% of adults in England were obese and an additional 36% were overweight [6]. Furthermore, 28% of children aged 2–15 years old were either overweight or obese [7]. The consequences of the negative health outcomes relating to obesity cost the NHS over £5 billion per year [8].

In 2015, the World Health Organisation (WHO) published guidelines recommending an intake of free sugars from sugary foods and drinks of no more than 10%, and ideally less than 5%, of total energy intake throughout the life course [9]. This 5% figure was endorsed by the UK Scientific Advisory Committee on Nutrition (SACN) [4] and Public Health England (PHE) [10]. However, free sugars currently account for 12–15% of total energy intake in the UK, approximately three times higher than the recommended limits [11, 12].

Several actions have been proposed to reduce sugar consumption in the UK, particularly in young people, including a 10–20% tax or levy on SSBs [10]. Analysis of the 10% sugar tax implemented in Mexico in January 2014 demonstrated the potential effectiveness of such policies to decrease sugar consumption, particularly in lower socio-economic households [13–15]. An econometric modelling study similarly predicted that a 20% SSB tax in the UK would produce a 1.3% reduction in adult obesity [16]. In March 2016, the UK government announced a soft drinks industry levy (SDIL), to be implemented in April 2018 [17–20], and similar measures have now been announced or introduced in some 20 countries, including Ireland, South Africa, Portugal, Spain, Estonia and parts of the USA [21].

Research suggests that the media could potentially play a powerful role in forming public perceptions, and thus potential acceptance, of public health policies [22–24]. Mass print media provides a wide platform for reaching the public, and has power in putting health issues on the public agenda and determining how those issues are framed [25, 26]. This framing process involves emphasising some aspects of a debate and downplaying others. Framing can thus include representations of the seriousness and causes of societal problems and the potential effectiveness of proposed solutions. Framing can therefore influence audiences’ perception and evaluation of different approaches to addressing public health problems [24]. As such, media framing of the issues around sugar and SSB consumption could have influenced public acceptance of upstream solutions, such as taxation.

Previous research found the consequences of sugar consumption to be an increasingly prominent topic in the UK media throughout 2014 [27]. However, the soft drink industry generally managed to avoid negative press with few calls for policy change such as enforced reformulation, clearer labelling or taxation and an emphasis on individual responsibility. The purpose of our study was to analyse how sugar and SSB policy debates were represented in UK newspapers at a time of heightened awareness due to key publications, campaigner activities and policy announcements including the UK Government’s SDIL; and compare these findings with earlier studies to inform future public health advocacy.

## Methods

We conducted quantitative newsprint content analysis using a method developed by Hilton and colleagues [23, 28–31] at the University of Glasgow’s Social and Public Health Sciences Unit to facilitate systematic recording of media content to understand framing [25, 26]. Typically, this research method involves: retrieving newspaper articles from a database; manually excluding insufficiently relevant results; constructing a coding frame designed to capture features of each article relevant to the research aims; systematically coding each article; and performing statistical analysis of the coded data. The research design of this study closely followed Elliott-Green and colleagues’ [27] prior content analysis of earlier media representations of SSBs to facilitate comparison of findings.

### Sample selection

We selected eight UK and three Scottish national newspapers, including each title’s Sunday counterpart. Newspapers with high circulation figures [32] were purposively selected, to ensure a sample of coverage that represented the variety of the UK and Scottish national newspaper audiences, using a typology employed in previous content analyses of UK newspapers [23, 33, 34]. This typology includes three newspaper ‘genres’: tabloid, middle-market and quality. Tabloid newspapers are politically diverse, with predominantly working-class readerships, and an informal tone. Middle-market newspapers are more serious in tone, have a predominantly older, middle-class readership, and are typically aligned with the political centre-right. ‘Quality’ newspapers are those formerly categorised as broadsheets, and are politically diverse, with predominantly middle-class readerships. The use of this typology ensured a sampling frame of newspapers diverse in terms of political alignment and the age and social class of their audiences.

A time period from 1^st^ April 2015 to 31^st^ November 2016 was chosen to capture the initial media debate prompted by the publication of WHO guidelines on sugar intake in March 2015 [9], the SACN report on carbohydrates in July 2015 [4], the PHE report calling for action on sugar consumption in October 2015 [10], and the Government consultation process on the SDIL which closed in October 2016 [19, 35].

### Database search and manual filtering

**Fig 1:**
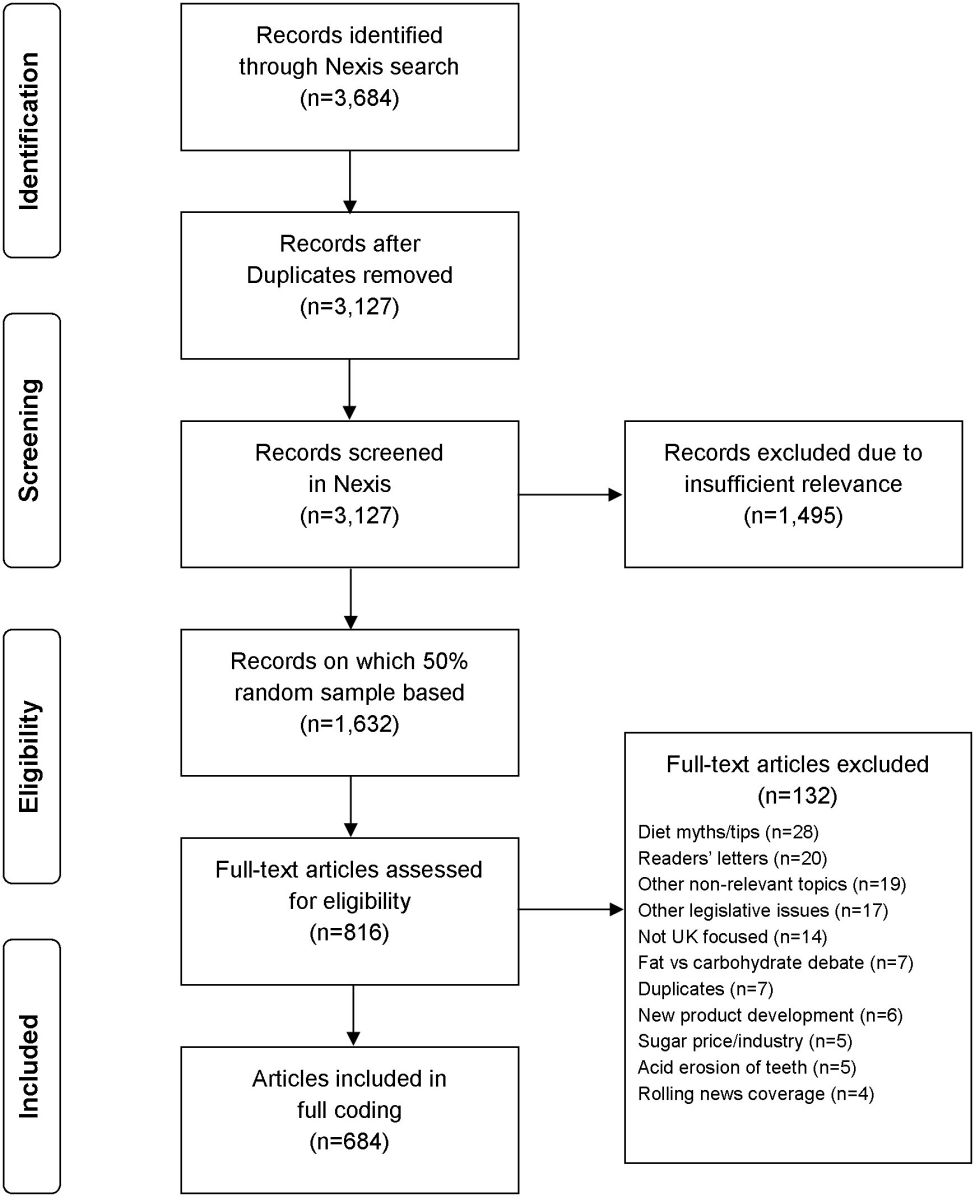
Flowchart summary of sampling process.

After identifying the sampling frame, we arrived at a final sample of relevant articles through a methodical process of: 1) searching for articles; 2) removing clearly irrelevant articles based on headlines; 3) extracting a random sub-sample of articles; and 4) assessing the full-text of articles for eligibility and excluding as appropriate (Fig 1).

We searched the Nexis database for articles that contained both three of more mentions of “sugar*” and one or more mention of the following: “beverage* OR soft drink* OR fizz* OR soda OR tax* or levy”. The headline of each article was read, and clearly irrelevant articles were excluded, while all other articles were exported for further scrutiny. To generate a representative sample that could be coded within the researcher time available, half of the exported articles were selected randomly and removed. Each of the remaining articles was read, and articles were **excluded** if: 1) less than 50% of their content related to sugar or SSBs; 2) they were short lead-ins to main stories elsewhere within the same issue (which also appeared in the sample); and/or 3) they were printed in publications’ letters, advice, TV guide, sport, weather, obituaries or review pages. Through this manual exclusion process, we arrived at the final research sample for coding.

### Coding and data analysis

A coding frame was developed to facilitate systematic quantitative recording of the relevant, explicitly stated content of each article (Supporting information S1 File). The coding frame closely followed that used by Elliott-Green and colleagues’ [27], covering: basic descriptive characteristics of articles; the topic(s) covered; articles’ slants towards sugar, SSBs and taxation; and different aspects of defining the problem of sugar, the problem’s drivers and the problem’s potential solutions. CHB tested and refined the coding frame based on a random 10% subsample of articles (n = 163), and a code descriptor document was developed to facilitate consistent interpretation of codes.

We used a combination of quantitative and qualitative methods to validate the coding frame. A process of double-coding and measurement of inter-rater reliability was employed to help identify any codes that were either interpreted incorrectly or applied inconsistently, and allow coding definitions to be refined. Working separately, CHB and LH double-coded a 10% sample (n = 163), and Cohen’s kappa coefficient was used to calculate inter-rater agreement for each code [36]. 66% of codes returned a kappa score >0.4, which is typically interpreted as ‘moderate’ agreement or better [37]. Where less than substantial agreement was identified (kappa <0.61), code definitions were discussed within the research team and the coding frame and descriptor document were revised as required.

For final coding, the 50% random sample of the articles was used to allow the capture of a representative sample of media coverage [38, 39]. The coding process entailed carefully reading the full text of each article, and, for each thematic code, recording whether the article overtly contained content relevant to that code; for example, a *Daily Mail (29*.*05*.*2015)* article that stated *“The Scientific Advisory Committee on Nutrition last week called on the nation to halve the current recommended intake of sugar to seven teaspoons a day to tackle the growing obesity and diabetes crisis”* was coded as both associating sugar with obesity and diabetes (either independently or through the mechanism of obesity). CHB completed final coding and coded data were entered into a spreadsheet and imported into SPSS Statistics 21 for analysis (Supporting information S2 File). Data are reported as counts and proportions throughout. Excerpts of text from articles are presented to illustrate quantitative data where appropriate.

## Results

### Overview of sample

The initial database search returned 3,127 articles, following the sampling and filtering process described in the methods section (Fig 1), the final sample comprised 684 articles. Table 1 lists their distribution by newspaper and region. Forty (5.8%) articles were printed on front pages.

**Table 1:** Distribution of articles by newspaper and region.

Region	Publication	Articles		Front page	
		n	%	n	%
UK (n = 606)	The Independent	121	17.7	13	1.9
	The Times & Sunday Times	112	16.4	10	1.5
	The Guardian & The Observer	108	15.8	0	0.0
	Telegraph & Sunday Telegraph	80	11.7	9	1.3
	Sun & Sunday Sun	77	11.3	2	0.3
	Daily Mail & Mail on Sunday	49	7.2	0	0.0
	Mirror & Sunday Mirror	33	4.8	0	0.0
	Express & Sunday Express	26	3.8	3	0.4
Scotland (n = 78)	Herald & Sunday Herald	36	5.3	2	0.3
	Scotsman & Scotland on Sunday	24	3.5	0	0.0
	Daily Record & Sunday Mail	18	2.6	1	0.1
	**Total**:	**684**	**100**	**40**	**5.8**

**Fig 2:**
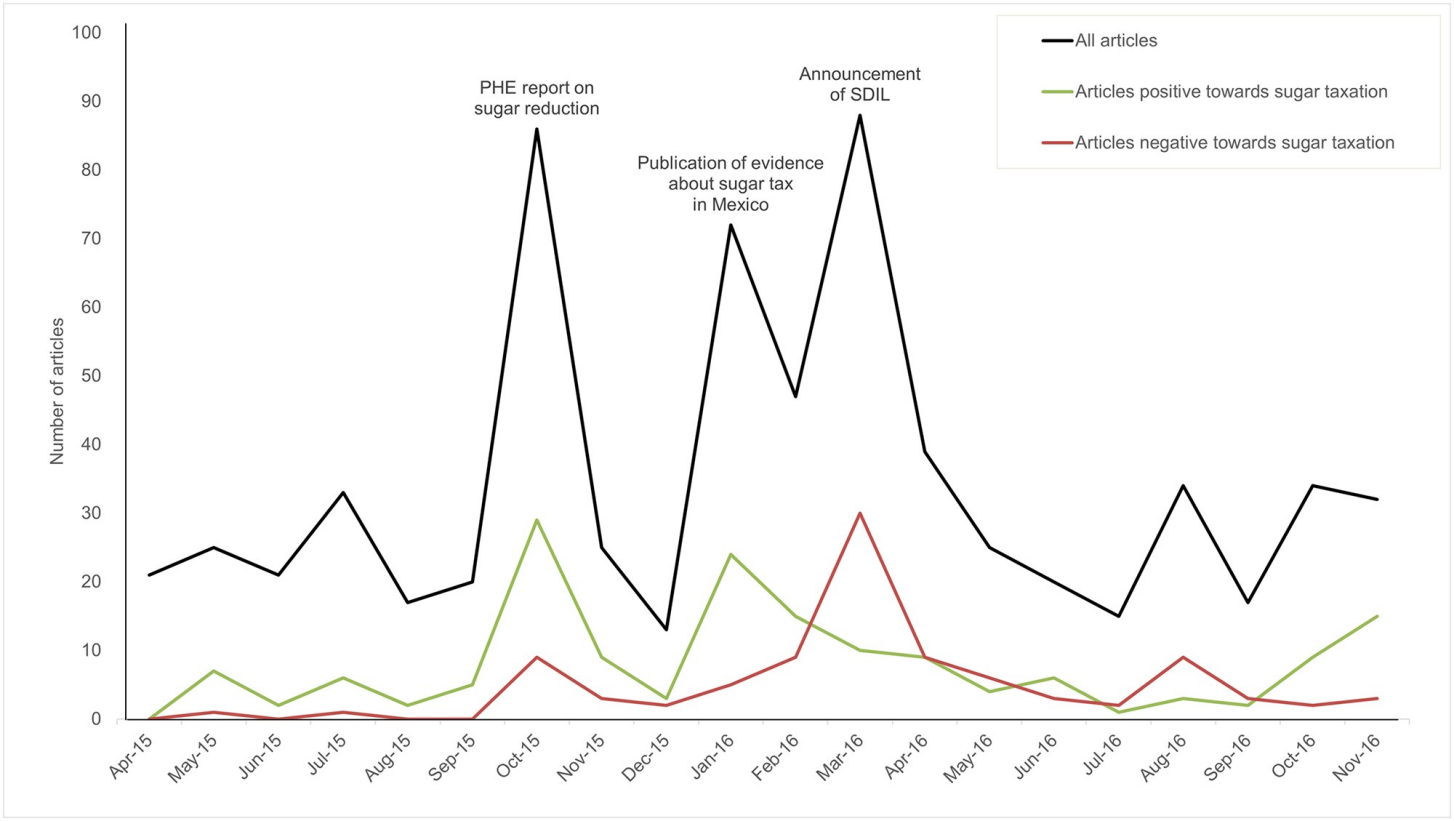
Frequency of articles over time and by stance towards sugar taxation. (Balance represents articles with an overall neutral slant on taxation).

Fig 2 illustrates the distribution of articles by month across the sample period, highlighting a period of elevated coverage between October 2015 and May 2016 (Fig 2). Peaks in reporting during this period coincided with the publication of the PHE report on sugar reduction in October 2015 [10]; the publication of early findings on the effectiveness of sugar tax in Mexico in January 2016 [14, 15]; and the UK Government’s announcement of a planned SDIL in March 2016 [18, 19].

### Articles’ slant towards taxation of SSBs

Articles’ slants towards taxation of SSBs were recorded. More articles were positive about taxation (n = 161, 23.5%) than negative (n = 97, 14.2%), but most were either neutral or exhibited no stance (n = 426, 62.3%). Fig 2 charts the frequency of articles coded as positive or negative towards taxation by publication month. Articles were largely positive about taxation during the first two peaks in coverage (in reaction to the PHE report and Mexican evidence respectively [10, 15], but negativity increased noticeably around announcement of the SDIL in March 2016 [19].

### Definitions of the sugar problem

**Fig 3:**
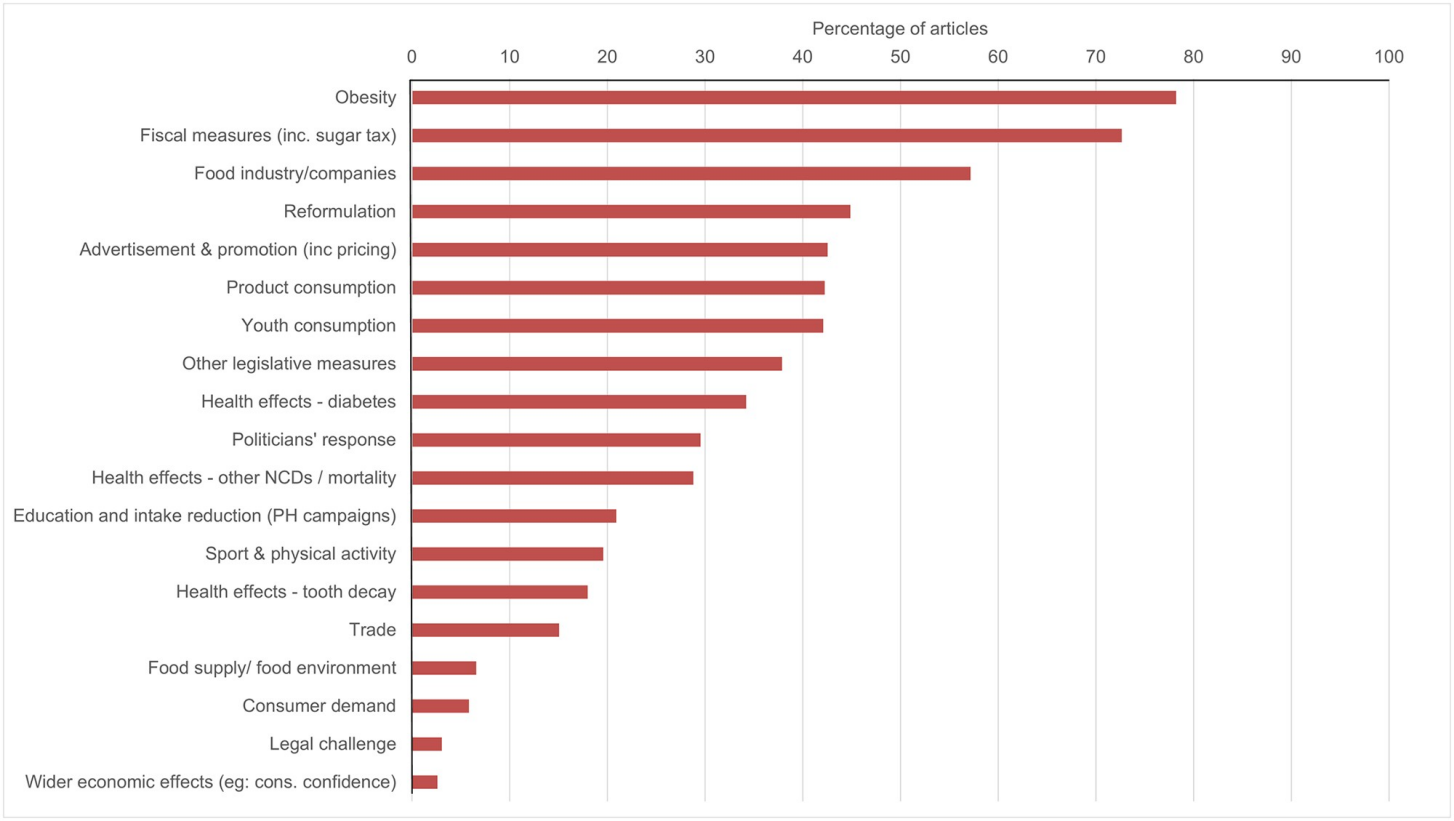
Proportion of articles discussing specific topics.

The frequencies with which specific topics were covered within articles are shown in Fig 3, illustrating the prominence of obesity (n = 535, 78.2%), fiscal measures (n = 497, 72.7%), the food industry (n = 391, 57.2%), reformulation (n = 307, 44.9%) and marketing (n = 291, 42.5%) (Fig 3). Most articles contained discussion of problems associated with sugar (either in general or in relation to SSBs) (n = 591, 86.4%).

Table 2 lists the frequencies of articles mentioning different aspects of the problem of sugar consumption, including: affected groups; sources of sugar; health consequences; wider societal consequences; problems specific to SSBs over other sources of dietary sugar; and the perspective that sugar consumption is not an important problem. Sugar was characterised as affecting young people and children in 273 (39.9%) articles, and as affecting old and young alike in a similar proportion (n = 275, 40.2%). For example, *The Express* (*10*.*09*.*2016*) focused on children, warning that *“children eat three times too much sugar”*, while a *Daily Mirror* editorial (*21*.*07*.*2015*) warned that *“nobody’s safe from sugar”*. Relatively few articles defined the problem in terms of gender (n = 5, 0.7%) or socio-economic status (SES) (n = 51, 7.5%). *The Times* (11.10.2016) identified both gender and socioeconomic differences in an article titled *“Affluent girls are more likely to become obese than boys”*, reporting on both a gendered *“weight gap”* among high-SES groups and higher predicted prevalence of obesity within low-SES groups.

**Table 2:** Articles’ definitions of problems associated with sugar and SSB consumption.

Theme	Total (n = 684)	
	n	%
Consumption is a problem specifically for		
Young people and/or children	273	39.9
Old and young alike / the general population	275	40.2
The problem varies by gender	5	0.7
The problem varies by socio-economic disadvantage	51	7.5
The sugar problem is particularly related to		
Specific types of product (e.g. energy drinks)	25	3.7
"Hidden" sugars in everyday products	102	14.9
Sugar consumption gives rise to		
Obesity or weight gain	481	70.3
Tooth decay	125	18.3
Diabetes	224	32.7
Other NCDs and mortality	192	28.1
Wider consequences of consumption include		
A burden on the National Health Service	149	21.8
A wider economic cost to society (e.g. business or worker productivity)	42	6.1
Sugar-sweetened beverages are particularly problematic because		
They are the single biggest source of sugar in people's diet	64	9.4
They have no nutritional value or health benefits, only "empty calories"	45	6.6
They do not satiate appetite or reduce calorie intake from food	11	1.6
Sugar consumption is not a problem		
The problem has been over-hyped / other issues to blame for obesity	71	10.4

The health impacts of sugar were primarily defined weight gain or obesity (n = 481, 70.3%), followed by diabetes (n = 224, 32.7%), tooth decay (n = 125, 18.3%) and other diseases such as heart disease (n = 192, 28.1%). For example, the *Observer* (*26*.*06*.*2016*) described sugar as “*a major dietary villain […] associated with obesity and other chronic health conditions such as type 2 diabetes and cardiovascular disease*”, and identified tooth decay as “*the most common reason for five- to nine-year-olds to be admitted to hospital*”. Consequently, 149 (21.8%) articles described sugar consumption as a burden on the NHS; *The Express* (*15*.*02*.*2016*) characterised high prevalence of type 2 diabetes prevalence as *“the time bomb facing NHS”*, while *The Times* (*5*.*01*.*2016*) labelled the same phenomenon a *“health crisis”* and an *“ever-growing cost to the NHS now estimated at £10 billion a year”*.

Articles that focused on SSBs as a source of problems characterised SSBs as being people’s single biggest source of dietary sugar (n = 64, 9.4%), having no nutritional value (n = 45, 6.6%), and not satiating the appetite, and therefore not reducing calorie intake from food (n = 11, 1.6%). A minority of articles (n = 71, 10.4%) suggested that problems of sugar consumption have been exaggerated. Typifying this, an article in *The Sun* (*21*.*05*.*2016*) titled *“Sham of sugar tax as sales plummet”* reported that soft drink sales had fallen “*without a sugar tax*”.

### Drivers of sugar consumption

Most articles (n = 465, 68.0%) contained some discussion of drivers of sugar consumption. Mentions of specific drivers were coded, and specific drivers were categorised as relating to either individuals, industry, society or government. Table 3 lists the specific drivers and the frequency with which each one was mentioned, as well as the categories to which those specific drivers were assigned. Most articles mentioned an industrial driver (n = 398, 58.2%), while 192 (28.1%) mentioned a governmental driver, 172 (25.2%) mentioned individual drivers and 62 (9.1%) mentioned societal drivers. While articles often mentioned drivers from more than one category, a minority of articles mentioned industrial drivers exclusively, without mentioning drivers from any other category (n = 156, 22.8%). Relatively few articles exclusively focused on societal (n = 3, 0.4%), governmental (n = 15, 2.2%) or individual (n = 29, 2.9%) drivers.

**Table 3:** Mentions of potential drivers of problematic consumption of sugar and SSBs.

Theme	Total (n = 684)	
	n	%
Failure of the individual		
Biological, genetic or psychological predisposition among individuals	64	9.4
Personal choice	76	11.1
Family background	49	7.2
Behaviour of mothers	3	0.4
Changing eating habits (e.g. increasing use of convenience foods)	38	5.6
Failure of industry		
Inadequate industry self-regulation, vested interests and lobbying	111	16.2
Inadequate or confusing product labelling	70	10.2
Production of "unhealthy" products (and lack of reformulation)	306	44.7
Advertising & marketing	165	24.1
Product pricing and pricing promotions	114	16.7
Insufficient ‘healthy’ alternatives provided by restaurants/catering outlets	20	2.9
Failure of society		
Abundance of fast food outlets (near schools / in high-deprivation areas)	14	2.0
Sugar consumption is ingrained in culture	33	4.8
Schools and education policy fails to support children’s 'healthy' diets	22	3.2
Failure of government		
Ineffective public health education	56	8.2
Food environment does not support 'healthy' choices	32	4.7
Inadequate formal regulation of food industry	46	6.7
Lack of fiscal measures to regulate industry	6	0.9
Politicians' behaviour (acting in interests of industry)	121	17.7

The most frequently mentioned specific industrial drivers were: producing (and failing to reformulate) “unhealthy” products (n = 306, 44.7%); advertising and marketing (n = 165, 24.1%); pricing and promotions (n = 114, 16.7%); and inadequate self-regulation (n = 111, 16.2%). A *Sunday Times* editorial (*7*.*02*.*2016*) encapsulated many of these, reporting that unhealthy food manufacturers have *“hidden behind sport to promote their products”*, and voluntary plans to reformulate products *“do not go far enough”*, while *“our kids are being directly targeted through the content they love”*. The most frequently mentioned governmental drivers were: politicians acting in the interests of industry (n = 121, 17.7%); ineffective public health education (n = 56, 8.2%); and inadequate regulation of the food industry (n = 111, 16.2%). The *Daily Mail* (*24*.*10*.*2015*) reported that *“health ministers have given food firms unprecedented access to the head of government”*, while the *Independent* described the government’s new childhood obesity strategy as *“watering down a raft of plans designed to slash Britain’s childhood obesity levels”* (*31*.*10*.*2016*), and characterised public health promotion campaigns as *“ineffective”* (*13*.*07*.*2015*).

### Potential solutions

Most articles (n = 588, 86.0%) mentioned one or more potential solution to the problem of sugar consumption. Each specific solution was assigned to one of four categories: individual, voluntary industrial action, societal and governmental (Table 4). Most articles mentioned a governmental solution (n = 473, 69.2%), while 318 (46.5%) mentioned voluntary industrial solutions, 190 (27.8%) mentioned individual solutions and 161 (23.5%) mentioned societal solutions. Articles often mentioned drivers from more than one category, but some articles only mentioned solutions from one category; 135 (19.7%) articles exclusively mentioned governmental solutions, while fewer mentioned industrial (n = 60, 8.8%), individual (n = 20, 2.9%) or societal (n = 2, 0.3%) solutions.

**Table 4:** Mentions of potential solutions to problematic consumption of sugar and SSBs.

Theme	Total (n = 684)	
	n	%
Individual responsibility		
Individuals should make better choices	169	24.7
Take early, individual preventative action with young children	57	8.3
Industrial voluntary responsibility		
Existing voluntary action is appropriate and should continue / increase	298	43.6
New ideas should be introduced by industry on a voluntary basis	54	7.9
Industry has role to play in public health education	95	13.9
Societal responsibility		
Better health policy in schools	101	14.8
Community-led initiatives	54	7.9
Cultural/attitudinal change	35	5.1
We need to better understand the role of sugar in diet and health	6	0.9
Governmental responsibility		
Better public health education and health promotion	108	15.8
Stronger legislation and regulation of industry	270	39.5
Taxation of sugar in general	185	27.0
Taxation of sugar-sweetened beverages, specifically	308	45.0
Improve food supply / food environment	34	5.0
Many stakeholders' responsibility		
Complex, coordinated set of measures involving all stakeholders	66	9.6
The solution lies beyond the issues of sugar and SSBs		
Single-nutrient approach insufficient; must also target fat, junk food etc.	76	11.1

Frequencies of mentions of specific solutions within the four categories are described in Table 4. The most frequently mentioned governmental solutions were: taxation of SSBs (n = 308, 45.0%); stronger industry regulation in general (n = 270, 39.5%); and general sugar taxation (n = 185, 27.0%). A *Daily Mail* article (*26*.*01*.*2016*) reported that the WHO described *“a tax on sugary drinks as one of nine urgent actions that governments should take to fight childhood obesity”*, while *The Sun* (*11*.*01*.*2016*) reported that *“PHE’s proposed rate of SSB taxation should be increased to 50%”* and an opinion piece in *The Guardian* (*11*.*04*.*2016*) suggested that *“it makes sense as well to regulate what kind of extraneous sugars make it into packaged foods”*. The most frequently mentioned industrial solution was continued or greater voluntary action (n = 298, 43.6%). A *Daily Telegraph* article (*26*.*05*.*2016*) written by a Coca-Cola executive described the corporation’s voluntary product reformulation and marketing activities, with which they *“seek to encourage more people […] to choose a no sugar drink”*, while the *Daily Mirror* (*23*.*01*.*2016*) reported that *“soft drink giants have vowed to help cut sugar by a fifth to tackle obesity”*.

As well as mentioning a range of specific solutions, 66 (n = 9.6%) articles highlighted the necessity for a coordinated set of measures from multiple sectors; *The Guardian* (*4*.*02*.*2016*) reported that *“the multi-faceted reasons behind obesity will not be dealt with by a blunt rise in cost”*, arguing that *“more complex proposals to help tackle the crisis include reformulation; reduced portion sizes; restriction on advertising and marketing to children of certain products high in fat*, *salt or sugar; promotions of healthier food ranges; and further voluntary proposals for cleared labelling*”, as well as highlighting the importance of education.

## Discussion

Newspaper coverage of sugar and SSB consumption, and potential fiscal measures targeted at reducing consumption, has become increasingly high profile. Peaks in coverage coincided with three key policy events: PHE’s report on sugar reduction [10], evidence of effectiveness of the Mexican sugar tax [14, 15], and the UK Government’s SDIL announcement [19]. Articles were predominantly favourable towards taxation as a solution, particularly around the first two peaks, but there was evidence of a surge in opposition coinciding with the SDIL announcement. Most articles presented sugar as a health concern (76.6%), and the risks were often associated with children (39.9%). The problem of ‘excessive sugar’ consumption was characterised as being driven by the food and drink industry (n = 58.2%) (specifically their production (44.7%) and marketing (24.1%) of ‘unhealthy’, sugary products), and primarily characterised as having governmental solutions (69.2%), though industrial voluntary responsibility was also mentioned frequently (46.5%).

Our research represents a rigorous analysis of the framing of the problem of excessive sugar consumption within a representative sample of UK national newspaper articles. It offers insight into how the issue, its drivers and its potential solutions were presented to public audiences during a period of heightened public scrutiny of sugar and SSBs. These findings contribute to understandings of media representations of sugar and SSBs and the public policy landscape, and may inform future public health advocacy and media engagement related to unhealthy commodities and their potential policy solutions. This study cannot make claims about how audiences interpreted the content analysed, or how the policy process was affected by those interpretations. However, it builds upon literature establishing the powerful influence that media framing of health issues can have [25, 30, 40–42] and the importance of understanding how the corporations that manufacture and market unhealthy commodities are represented in the media [43, 44]. Furthermore, it also builds upon Elliott-Green and colleagues’ earlier analysis of UK newspaper representations of SSBs [27].

Our findings can be compared directly with Elliott-Green and colleagues’ analysis of public health advocacy versus pro-industry messaging within UK newspaper coverage of SSBs in 2014 [27]. In line with escalations in the policy debate, our analysis indicates coverage of sugar and SSBs continued to increase in 2015–16, illustrating the evolution of sugar from a relatively low-profile issue to frequent, often front-page, news. Furthermore, comparison of the two time-periods illustrates a change in narrative focus from defining the problem to discussing solutions. Articles in both periods shared a focus on obesity and health impacts, but differed somewhat in their representations of solutions. While articles about SSBs published in 2014 identified individual-level solutions more frequently than policy solutions, the articles in our sample gave more focus to governmental solutions, such as taxation and regulation of industry, and the ‘industry-friendly messaging’ identified in 2014 was less evident in our sample. Our findings thus complement research into debates about other unhealthy commodities. The stark rise in articles predominantly opposed to SSB taxation contemporaneous with the announcement of the SDIL echoes late ‘surges’ in opposition to tobacco control identified prior to elections and policy events [45, 46].

This research illustrates a recent media debate that was largely favourable to sugar reduction policy specifically, and to evidence-based public health advocacy more generally. The peaks in coverage demonstrate how public health reports and evidence can stimulate media agenda setting, and public health advocates will welcome the overwhelming representation of sugar and SSBs as a society-wide health problem, primarily driven by the food and drink industries, and best addressed through policy measures. While many articles identified the need for individuals to make healthier choices, they were outnumbered by articles framing the problem as driven by industrial and environmental phenomena. Consistent with political science theory, this may raise public awareness of the commercial determinants of health, in turn influencing public attitudes to policy solutions [47]. However, given that forms of voluntary industrial action were often cited as solutions, public health policy advocates’ task of promoting the appropriateness and necessity of governmental solutions is not finished. As such, ongoing media engagement may need to focus on the potential benefits of the ‘nanny state’. Acceptance of this may be a prerequisite for successfully addressing the problems of sugar and SSB consumption, for which ‘downstream’ interventions such as voluntary industry responsibility pledges have been found wanting [48, 49]. Furthermore, while the frames constructed by newspaper articles were predominantly favourable to public health advocacy, and supportive coverage outnumbered oppositional coverage, public health stakeholders should be aware of the predominance of oppositional articles around the announcement of the SDIL; a surge of opposition that a coordinated campaign of media advocacy might have mitigated.

This study’s findings are subject to a number of limitations. Firstly, while double-coding and inter-rater agreement were used to validate the coding instrument to foster consistency of interpretation and application, the final dataset was not double-coded, such that we can present no statistical measure of consistency of coding. However, the rigorous double-coding of a subsample of articles, and continued dialogue between both coders during that process, ensured that the final coding instrument was validated. Thus facilitating consistent interpretation and application of codes in final coding, within the limitations imposed by the inherently interpretive process of coding text content [50, 51]. Secondly, our findings reflect commentary exclusively presented in newsprint and cannot be generalised to broader media, particularly social media sources. Finally, the focus on manifest content analysis means that we may have over-looked subtleties in the debate, especially the nuanced way arguments representing multiple codes were constructed by stakeholders in supporting or opposing a point of view. These limitations notwithstanding, this is a comprehensive, robust study of newsprint representations of the policy debate around SSB taxation and the SDIL, which brings up to date earlier work in the area in both the UK and United States [27, 52].

Development and support for public health policy in relation to food and drink has been portrayed as problematic, in a way that is not the case for other commodities associated with ill health such as alcohol and tobacco [53, 54]. Further research is needed to explore the complexities of food and nutrition policy formulation. For example by undertaking latent content analysis of stakeholder argumentation to identify direct lobbying of the public by both proponents and opponents of such policy measures. It would also be useful to extend the analysis of similarities and differences across so called “unhealthy commodity industries”, to understand why food policy is regarded differently and thus formulate strategies to promote policy support.

In conclusion research across different perspectives and contexts, (including the media), thus has a potentially important role. It can inform the public health policy debate process, and thus equip stakeholders to better advocate for future evidence-based public health policies.

## Supporting information

S1 File Detailed coding frame used to analyse final sample of newspaper articles. (XLSX)

S2 File Excel spreadsheet of data extracted and coded from articles. (XLSX)
